# Control of Ochratoxin A Production in Grapes

**DOI:** 10.3390/toxins4050364

**Published:** 2012-05-14

**Authors:** María Lorena Ponsone, María Laura Chiotta, Juan Manuel Palazzini, Mariana Combina, Sofía Chulze

**Affiliations:** 1 Departamento de Microbiología e Inmunología, Facultad de Ciencias Exactas, Físico, Químicas y Naturales, Universidad Nacional de Río Cuarto, Ruta Nacional 601, (5800) Río Cuarto, Córdoba, Argentina; Email: lponsone@exa.unrc.edu.ar (M.L.P.); mchiotta@exa.unrc.edu.ar (M.L.C.); jpalazzini@exa.unrc.edu.ar (J.M.P.); 2 Instituto Nacional de Tecnología Agropecuaria (INTA), Luján de Cuyo, Mendoza, Argentina; Email: mcombina@mendoza.inta.gov.ar

**Keywords:** Ochratoxin A, prevention strategies, fungicides, biological control

## Abstract

Ochratoxin A (OTA) is a mycotoxin commonly present in cereals, grapes, coffee, spices, and cocoa. Even though the main objective of the food and feed chain processors and distributors is to avoid the extended contamination of plant-derived foods and animal feeds with mycotoxins, until now, complete OTA removal from foods and feedstuffs is not feasible. Prevention through pre-harvest management is the best method for controlling mycotoxin contamination. However, in the case that the contamination occurs after this stage, the hazards associated with OTA must be managed through post-harvest strategies. Due to the increasing number of fungal strains resistant to chemical fungicides and the impact of these pesticides on the environment and human health, maximum levels of chemical residues have been regulated in many products. Alternative methods are necessary to substitute or complement treatments with fungicides to control fungi under field or storage conditions. Yeasts are considered one of the most potent biocontrol agents due to their biology and non-toxic properties. Epiphytic yeasts are the major component of the microbial community on the surface of grape berries and they are evolutionarily adapted to this ecological niche. Nowadays, several yeast species included in different genera are considered as potential biocontrol agents to control both, growth of ochratoxigenic *Aspergillus* species and OTA accumulation.

## 1. Introduction

Ochratoxin A (OTA) is a mycotoxin commonly present in cereals, grapes, coffee, spices, cocoa and their processed products [1]. The toxin is produced by several species of the *Aspergillus* and *Penicillium* genera. OTA in cereals is mainly produced by *P*. *verrucosum* while OTA in grapes, coffee and cocoa is mainly produced by *A*. *carbonarius* [2]. In the last decade, OTA has received increased attention worldwide because of its hazard to human and animal health [3]. Due to this toxicity, the International Agency for Research on Cancer (IARC) has classified OTA as a group 2B carcinogen [4]. OTA can contaminate a wide variety of foodstuffs and maximum permitted levels have been established by the EU and other countries to reduce the risk of human exposure [5]. 

The critical factors that affect fungal growth during farming, harvesting and storage are temperature, moisture content and the period that the commodity remains under adverse conditions. Mycotoxin biosynthesis is also affected by a wide range of factors, broadly classified into physical, biological and chemical, and by interactions involving these factors. Temperature, and humidity are key factors that interact to affect mycotoxin biosynthesis. The knowledge of the factors involved in OTA production and their interaction will allow the prediction and prevention of OTA contamination [6,7,8]. Also it is possible to detect OTA without the presence of the ochratoxigenic species since processing or environmental changes can inactivate the fungal spores but not alter the toxin that remains in the substratum [9] 

Even though the main objective of the food and feed chain providers is to avoid the extended contamination of plant-derived foods and animal feeds with OTA, until now, complete OTA removal from foods and feedstuffs is not feasible [10]. Many efforts are being made to achieve OTA reduction. Prevention through pre-harvest management is the best method for controlling mycotoxin contamination [9,11]. However, if contamination occurs after this stage, OTA hazards must be managed through post-harvest strategies. The methods currently employed to prevent OTA contamination in grapes are reviewed.

## 2. Chemical Control of OTA Production: Fungicides and Antioxidants

Ochratoxigenic species can colonize damaged parts of plants. Therefore, it is logical to protect grapes from mechanical and insect damages. Although damaged grapes can be removed, inoculum sources such as weeds, agricultural residues or dirty farming materials also have to be minimized to reduce contamination [10,12] 

The use of chemical compounds is a very attractive strategy to prevent mycotoxin production [13]. In the case of pesticides, the implications for mycotoxin production need to be considered. It has been reported that some fungicides influence positively or negatively OTA production [14]. Lo Curto *et al*. [15] observed that application of some pesticides such as Azoxystrobin (a strobilurin derivative) or Dinocap (a dinitrophenyl derivative) in combination with sulfur effectively decreased OTA content in wines. In contrast, it was observed that pesticides such as Carbendazim and Chorus were ineffective in controlling sour rot caused by *Aspergillus* section *Nigri*. However, the application of another pesticide, Switch, led to a significant reduction in the incidence of black aspergilli on grapes. The fungicide Switch contains cyprodinil and fludioxonil which belong to the pyrimidine and pyrrolnitrin classes of fungicides, respectively. Since the fungicide Chorus contains cyprodinil and was ineffective against aspergilli, it was concluded that fludioxonil was the active ingredient of Switch [16]. The observation that fludioxonil can be used against black aspergilli is not surprising since pyrrolnitrin was previously found to be effective against black aspergilli [17]. In another study carried out in France [18], the fungicides Switch, Scala (containing the pyrimidine fungicide pyrimethanil) and Mikal (containing fosetyl-Al and the dicarboximide folpel) were effective for lowering fungal colonization and OTA content in wines. Moreover, in vitro studies done by Belli *et al*. [19] with 26 fungicides showed that fungicides that stopped *A*. *carbonarius* growth also inhibited OTA synthesis. In general, fungicides that contained copper or strobilurins reduced both, growth and OTA production, contrary to sulfur fungicides. Among the fungicides that inhibited *A*. *carbonarius* growth in synthetic medium, cyprodinil seemed to be the most effective active ingredient to stop fungal growth when reduced doses were tested. When these fungicides were tested on grapes, the effect was similar to that observed on synthetic medium [19]. It is important to remark that fungicides must be applied with care since some of them, such as carbendazim, have been found to reduce fungal flora but stimulate OTA production [15]. 

Another strategy to reduce fungal growth and mycotoxin production is the use of antioxidants such as vanillic acid or 4-hydroxybenzoic acid [20] and essential oils extracted from plants such as *Thymus vulgaris* or *Aframomum danielli* [11,21], which affected both, fungal growth and OTA synthesis. From a human health perspective, the use of antioxidants as antimicrobial agents is allowed by the US Food and Drug Administration (FDA) and regarded as safe (GRAS) chemicals. Even though these antioxidants have not been tested on dried vine fruits, some studies have shown that these compounds have a protective action in food since they could maintain organoleptic properties [22,23,24,25]. This could be an interesting option for commodities that requires long storage periods, such as dried vine fruits. 

Trans-resveratrol (3,5,4-trihydroxystilbene) is an antioxidant compound naturally produced in a huge number of plants, including grapes, being the major component of the phytoalexin response of the plant. It accumulates in vine leaves and grape skin in response to various fungal infections, UV radiation, or chemicals [26,27,28], and it has been found in wines in varying concentrations depending on viticultural and enological practices [29]. 

Analytical interest in trans-resveratrol was attributed to its natural pesticide properties. A recent study showed that trans-resveratrol is fungitoxic at physiological concentrations against *B*. *cinerea* [30]. However trans-resveratrol has also been proven to enhance the resistance of vine plants to other pathogens, such as *Plasmopara viticola* [31], *Phomopsis viticola* [32], or *Rhizopus stonifer* [33]. Recently, Perrone *et al*. [34] detected a positive correlation in wine samples from southern Italy, between levels of OTA and total stilbenes as well as between OTA and total resveratrols and between OTA and total piceids, suggesting that OTA production stimulates stilbenes synthesis. This rather unspecific antifungal character and the selective accumulation of trans-resveratrol in grape skin make it a good candidate as a natural pesticide against pathogen attack for improving the natural resistance of grapes to fungal infection. In addition, because of its antioxidant properties, trans-resveratrol can also have positive effects on fruit conservation during storage. As a consequence, both endogenous enhancement and exogenous application could be exploited to reduce grape spoilage. 

It is important to take into account that the determination of optimal application doses of antifungal substances is very important, because high levels could produce undesirable effects on the grain organoleptic properties and on its processed products, causing a negative economic impact. On the other hand, sub-inhibitory doses together with inadequate distribution of chemicals, especially at low a_W_ values, could cause fungal sporulation, growth and secondary metabolism stimulation, increasing mycotoxin production [35].

## 3. Biocontrol as an Ecofriendly Strategy

Although chemicals have been commonly used to reduce fungal proliferation and mycotoxin production under field conditions, nowadays a strict legislation about their use has been established in the European Union, due to the increasing number of resistant fungal strains and the impact of fungicides on the environment and human health [36]. Maximum residue levels of pesticides have been regulated in many products, including grapes [37]. Therefore, alternative methods are necessary to substitute or complement fungicide treatments to control toxigenic fungi at pre- and post-harvest stages.

Biological control using antagonist microorganisms has been proposed for a long time as a good option to control plant pathogens [38]. One of the advantages of biocontrol is that it could be used together with fungicides reducing their levels in order to decrease fungal growth [39,40,41]. 

Yeasts are considered one of the most potent biocontrol agents due to their biology and non toxic properties [42]. The mechanism most probably involved in filamentous fungi biocontrol by yeast is competition. Competition among microorganisms for essential factors, such as nutrients and space, is expected to have a dramatic effect on the secondary metabolism of filamentous fungi [41,43]. On the other hand, parasitism and production of fungal growth inhibiting compounds have been also described [42,44]. Nowadays, several yeast species included in different genera are considered potential biocontrol agents towards ochratoxigenic *Aspergillus* [45,46,47,48]. Epiphytic yeasts are the major component of the microbial community on the surface of grape berries and they are evolutionarily adapted to this ecological niche [49]. Yeasts, indeed, can be effective biocontrol agents due to their capacity to colonize grapes and compete for space and nutrients with other microorganisms [50]. In this direction, Zahavi *et al*. [48] selected two antagonistic yeast isolates, *Candida guillermondii* and *Acremonium cephalosporium*, and showed that they were efficient in reducing decay caused by *Botrytis*, *Rhizopus* and *Aspergillus*. In addition, Bleve *et al*. [45] showed that *Issatchenkia orientalis* have a strong antagonistic action against ochratoxigenic species; moreover, they also indentified an isolate of *Metschnikowia pulcherrima* and an isolate of *Candida incommunis* that also significantly reduced *A*. *niger* and *A* .* carbonarius* growth both *in vitro* and *in situ*. In another study carried out by Dimakopoulou *et al*. [46], it was demonstrated that one strain of *Aureobasidium pullulans* was effective in reducing sour rot infection, *A* .* carbonarius* presence on berries at harvest and OTA contamination in must. Recently, Ponsone *et al*. [47] described two epiphytic strains of *Lanchancea thermotolerans* that were able to control the growth and OTA accumulation of ochratoxigenic fungi both “*in vitro*” and “*in situ*”. The data reported until now indicate that the yeasts that occur naturally on grapes, are promising ecological fungicides, because they can survive and colonize grape berries in the vineyards and also maintain the equilibrium of the natural environment. 

In order to prevent a pathogen from establishing itself on the plant, it is important to have the biological control agent on the fruit surface before the arrival of the propagules of the pathogen [51]. This can be achieved either through frequent applications, or by using strains that can survive on the field [52]. 

The future use of biological control agents for controlling ochratoxigenic fungi and ochratoxin production will depend on the cost production and the field effectiveness of the formulated product. Therefore, the optimization of biocontrol efficacy also depends on survival and colonization of biological control agents in wounded and unwounded fruit surfaces and in the presence of low quantities of fungicides applied separately or in combination with microbial antagonists. Moreover, the widespread diffusion of fungal pathogens resistant to fungicides used for a long time in the field and/or in packinghouses (e.g., benzimidazoles), has led to the need of assessing the compatibility and efficacy of biological control agents with new and recently developed fungicides [53]. 

The application of biocontrol agents during vintage should not affect the organoleptic characteristics of the harvested grapes. To avoid the changes on the harvested grapes the application of CuSO_4_ can be done 20 days prior to harvest. For grapes used for wine production, a normal practice in the vinification process is the addition of sulfur dioxide (sulfiting step), which leads to the inhibition of indigenous yeasts ensuring an effective seeding with the chosen commercial yeast strain for the alcoholic fermentation. Also, during the final step of wine production, a racking step takes place that eliminates the solids present in the wine, including yeast cells, yeast lees, and small solids [54].

In conclusion, an integrated strategy based on the combination of biological control agents with natural compounds or reduced dosage of fungicides appears to be one of the most reliable options for large-scale utilization of microbial antagonists in the control of ochratoxigenic fungi and reduction of the entry of OTA to the food chains [55,56]. 
